# Highly pathogenic avian influenza in Canada: Spatial analysis of ecological hot and cold spots and environmental drivers

**DOI:** 10.1016/j.onehlt.2026.101630

**Published:** 2026-10-22

**Authors:** Zahra Movahedi Nia, Nicola Luigi Bragazzi, Murray Gillies, Emma Gardner, Jude Dzevela Kong

**Affiliations:** a Artificial Intelligence & Mathematical Modeling Lab (AIMMLab), Dalla Lana School of Public Health, University of Toronto, 155 College St, Room 500, Toronto, ON M5T 3M7, Canada; b Global South Artificial Intelligence for Pandemic and Epidemic Preparedness and Response Network (AI4PEP), Toronto, ON, Canada; c Africa-Canada Artificial Intelligence and Data Innovation Consortium (ACADIC), Toronto, ON, Canada; d Department of Mathematics, York University, Toronto, ON M3J 1P3, Canada; e Department of Food and Drugs, University of Parma, 43125 Parma, Italy; f Canada Animal Health Surveillance System (CAHSS), Animal Health Canada, Elora, ON N0B 1S0, Canada; g Institute of Health Policy, Management and Evaluation (IHPME), University of Toronto, Toronto, ON, Canada; h Department of Mathematics, University of Toronto, Bahen Centre for Information Technology, Room 6291, 40 St. George Street, Toronto, ON M5S 2E4, Canada; i Munk School of Global Affairs & Public Policy, University of Toronto, Toronto, ON, Canada

**Keywords:** Anselin local Moran's-I, Canada, Generalized additive models (GAM), Gets Ord, Highly pathogenic avian influenza (HPAI), Random Forest, SHapley additive exPlanations (SHAP), XGBoost

## Abstract

The number and magnitude of Highly Pathogenic Avian Influenza (HPAI) outbreaks have increased globally in recent years, placing substantial burdens on public health and causing significant economic losses to the poultry industry. Identifying spatial hotspots and key environmental drivers of HPAI is essential for improving surveillance efficiency and enabling targeted prevention strategies. This study aimed to identify spatial hotspots and cold spots of HPAI in Canada and determine the most influential predictors of HPAI occurrence at both national and local levels. Subdivision-level data were compiled from multiple sources, including reported HPAI cases in wild birds, waterfowl population density, poultry premises, weather and air quality indicators, vegetation cover, elevation, gross primary production, water bodies, and wildfire occurrence. Spatial cluster analyses using Getis-Ord Gi* and Anselin Moran's I were conducted to detect hotspots and cold spots and generate risk maps. Generalized Additive Models (GAM), XGBoost, and Random Forest Regression (RFR) were compared using R-squared score, root mean square error, and mean absolute error, and Shapley Additive exPlanations were applied to the machine learning models to quantify predictor importance. Consistent HPAI hotspots were identified in southwestern British Columbia, central and southern Alberta, southern Ontario, and the Atlantic provinces, while cold spots were observed in several central and northern regions. Hotspots of HPAI in domestic birds coincided with hotspots in wild birds, indicating that poultry and bird farms located in these areas are at higher risk of infection. GAM achieved the highest cross-validation accuracy, with XGBoost slightly outperforming RFR. Across all models, waterfowl population density, poultry premises, vegetation metrics including gross primary production, and air quality emerged as the most influential predictors. These findings provide actionable insights to support risk-based HPAI surveillance, targeted biosecurity interventions, and improved resource allocation in Canada.

## 1 Introduction

Highly Pathogenic Avian Influenza (HPAI), particularly the H5N1 subtype, has emerged in recent years as a serious concern to domestic and wild birds, and mammals, including humans, worldwide. It has devastated farms and poultry industries, disrupted the food trade, and caused enormous financial losses [1], [2]. HPAI outbreaks have spread along migratory bird routes, reaching 108 countries across five continents as of December 2024 [3].

In Canada, the H5N1 subtype was first confirmed in wild birds on November 26, 2021, followed by detection in domestic flocks on December 9, 2021 in Newfoundland [4], [5]. By early 2022, outbreaks had been reported in other provinces such as Nova Scotia, Ontario, and Alberta. HPAI continues to cause significant impacts to domestic poultry flocks and vulnerable wildlife species, and remains a pathogen with pandemic potential [6]. During the recent outbreaks caused by the Goose/Guangdong lineage H5N1 clade 2.3.4.4b, mammals are being increasingly infected with H5N1, raising the concern that the virus may adapt to infect humans more easily [7], [8].

Wild waterfowl and shorebirds serve as natural reservoirs, and HPAI can spill over into domestic poultry either through direct contact, or indirectly via environmental contamination, leading to devastating economic losses. Because the emergence and spread of avian influenza are heavily shaped by ecological and environmental drivers [9], understanding these drivers is crucial to anticipating and mitigating outbreaks. Global risk models have, for instance, identified vegetation, and land cover types as significant predictors of H5N1 occurrence in wild birds [10], [11].

Identifying high-risk habitats and environmental conditions enables the development of predictive risk maps, which can guide targeted monitoring programs, early-warning systems, and farm-level biosecurity interventions. Risk-based approaches that integrate ecological heterogeneity are particularly important in a country with a huge land mass and sparse human population density as Canada, where accessibility and resource allocation for wildlife and environmental surveillance are a major challenge. Ultimately, disentangling the role of environmental drivers provides the foundation for evidence-based policy and resource allocation, reducing the impact of HPAI outbreaks on wildlife, agriculture, and human populations alike [12].

An extensive body of research has investigated environmental factors linked to HPAI occurrence worldwide. However, relatively few studies have addressed the Canadian context, despite the country's position along major migratory flyways and its substantial poultry industry. This gap underscores the need for spatially focused analyses of HPAI in Canada to better understand local risk factors and inform targeted surveillance and control strategies. Liu et al. [13] applied logistic regression to identify potential risk factors for avian influenza in wild and domestic birds in Denmark during the 2020–2021 outbreaks. Their analysis indicated that both bird order and proximity to wetlands were associated with the occurrence of avian influenza. Gierak and Śmietanka [14] employed generalized linear and non-linear models including logistic regression, classification trees, and random forests to identify potential risk factors for HPAI in Poland. Their findings highlighted turkey, goose, and duck densities as the most influential predictors.

Azat et al. [15] applied linear regression to identify HPAI hotspots in Chile, revealing that landscape-scale factors, particularly temperature range, bird species richness, and human footprint, were associated with outbreak occurrence. Gilbert et al. [16] employed logistic regression to investigate risk factors for avian influenza in Vietnam and Thailand. Their analysis showed that HPAI outbreaks were consistently associated with key environmental variables, duck abundance, human population density, and rice-cropping intensity, while exhibiting a comparatively weaker association with chicken numbers. Wang et al. [17] used XGBoost to discover that the density of poultry farms plays a crucial role in determining outbreak risks in China. Their results show that climatic factors like temperature, seasonality, and moisture regimes also interact with habitat and species behavior to modulate both viral persistence outside hosts and opportunities for transmission.

In the Canadian context, surveillance following the 2021 H5N1 incursions has shown that detections have occurred in all provinces and territories, with greater incidence along major flyways, especially Atlantic and Central provinces [18]. The prevalence of HPAI in wild birds is significantly lower in the southeastern Arctic region of Canada compared to the Atlantic region [19]. Giacinti et al. [20] applied spatial scan statistics to identify clusters of high and low HPAI occurrences in wild birds across Ontario. Their results show that regions with dense human populations, abundant wild birds, and numerous poultry facilities overlap with areas of HPAI infection. Kahlon [21] found that the mean minimum daily temperature has a complex and non-linear relationship with the prevalence of the avian influenza virus in migratory birds. However, none of these papers have studied the importance of climate determinants such as vegetation, elevation, water proximity, wildfire information, total rainfall, and air quality, in H5N1 occurrences, in Canada. In the present study, we address this gap by incorporating these variables into a spatially explicit analysis and presenting novel empirical evidence on their association with recent Canadian outbreaks. Since very few cases of HPAI have been detected in the territories (Yukon, Nunavut, Northwest Territories), these regions were excluded from our analysis.

Our contribution to this field is five-fold. First, we employ spatial statistical methods to detect hot and cold clusters of HPAI occurrence, producing risk maps that highlight areas of elevated vulnerability and provide a valuable decision-support tool for surveillance, biosecurity, and outbreak response planning. Second, we examine the influence of climate and environmental factors, such as vegetation, weather, air quality, elevation, water index, and wildfire information, on HPAI occurrence, which has not previously been investigated for Canada. Third, we conduct our analysis at a granular geographic scale (census subdivision), enabling high-resolution insights that support targeted surveillance and intervention strategies, optimizing the use of limited resources. Fourth, we analyze spatial patterns at both national and local scales, capturing nationwide trends while identifying region-specific dynamics that may be obscured in aggregated analyses. Finally, we contribute a curated dataset of ecological and environmental variables for Canada, providing a novel resource that supports not only this study but also future research on HPAI and other environmentally influenced infectious diseases.

By identifying environmental and climatic determinants of HPAI at high spatial resolution, our study provides actionable insights for public health authorities and the poultry industry. Understanding hotspots of elevated risk enables the timely implementation of targeted interventions, enhancing outbreak control, minimizing the need for widespread culling, and reducing potential trade restrictions. Moreover, these findings support proactive surveillance and biosecurity planning, ultimately helping to mitigate the ecological, economic, and public health impacts of HPAI in Canada.

## 2 Methods

### 2.1 Data sources

Cases of HPAI in wild birds were obtained from the HPAI Wildlife Dashboard [22], which reports confirmed detections in wild birds across Canada, from November 4th, 2021 to May 1st, 2025. The dataset includes the census subdivision of each sample and its collection date, with all cases confirmed by the Canadian Food Inspection Agency's National Centre for Foreign Animal Disease (NCFAD). A census subdivision is the general term used by Statistics Canada to describe municipalities or areas treated as municipal equivalents for statistical purposes; these are administrative units nested within census divisions and represent the smallest standard geographic areas for which consistent demographic and agricultural data are available [23].

Due to the very few numbers of HPAI cases in the territories (i.e. Yukon, Northwest Territories, and Nunavut), these regions were excluded from our analysis. In addition to wild bird data, confirmed cases of HPAI in domestic poultry were obtained from the World Animal Health Information System (WAHIS) dashboard maintained by WOAH (World Organisation for Animal Health) [24]. The WAHIS dataset provides event-level reports of infected domestic flocks, including the geographic coordinates (latitude and longitude) of each affected premises and the start and end date of the outbreak. These data were used to assess the influence of wild bird infections on poultry and bird farms. Only outbreaks of domestic poultry events reported between November 4th, 2021 and May 1st, 2025 were included to match the temporal scope of the wild bird data.

The number of poultry production premises per subdivision was approximated by summing the number of farms reporting for all poultry production types (i.e. broilers, roasters, Cornish, and turkeys) from Statistics Canada [25]. Because some farms may produce multiple poultry types, this value represents an upper-bound estimate of the number of poultry farms. Wild bird population data were obtained from Project FeederWatch of the NatureCounts platform [26]. This project records observations of multiple bird species across North America (Canada, the USA, and Mexico), from November through April each year, exactly overlapping with the peak period of HPAI cases in Canada [18], [20]. The dataset provides both the date and geographic coordinates of each observation. We filtered the dataset for Canada, and waterfowl only. A complete list of the waterfowl species used for filtering is provided in Supplementary File 1. Next, the annual totals of waterfowl for each subdivision were averaged over the years 2021–2024.

Several climate and environmental indicators were collected from various sources. Weather data including mean minimum daily temperature and total rainfall were collected from Open-Meteo [27], [28], [29], [30], [31] at an approximately 333 Km resolution. These data were then spatially interpolated in ArcGIS pro using the ordinary kriging method [32] to transform it into a 232 m resolution. This 232 m resolution was chosen because it has sufficient granularity to ensure at least one value within each subdivision. Then, for each subdivision, minimum daily temperature values were averaged, and total rainfall values were summed for each year, and averaged over 2021–2024.

Maximum Air quality parameters, namely, carbon-monoxide (CO), nitrogen-dioxide (NO2), and methane (CH4), were collected from the Sentinel-5 mission of Google Earth Engine (GEE) [33]. These data were transformed from a 1113.2 m resolution, to a 232 m resolution using the GEE resampling and reprojecting attributes. The data were then aggregated by taking the maximum value of each parameter within each subdivision. Similarly, wildfire information which was retrieved from GEE FIRMS [34] collections, was converted from 1000 m resolution to 232 m resolution, and then maximized over the subdivisions. Weather, air quality, and wildfire data were collected on an annual basis for years 2021 to 2024, and then averaged over the years. Likewise, Gross Primary Productivity (GPP), representing the total carbon captured by vegetation within the ecosystem, was obtained from the MODIS dataset (MOD17A3HGF.061) via Google Earth Engine [35]. The original 500 m resolution data were resampled and reprojected to 232 m, averaged across the years 2021–2024, and subsequently minimized over each subdivision.

Other environmental variables collected from GEE are static and do not change over a four-year period. Elevation was obtained from GEE Terrain Elevation Data [36] at 231.92 m resolution. From the MODIS collections [35], two additional datasets were used: (1) vegetation (MOD44B.006) at 250 m resolution, i.e. tree coverage, non-tree vegetation, and non-vegetation, and (2) water bodies (MOD44W.006) at 250 m resolution. Elevation, vegetation, and water bodies were maximized over the subdivisions. These aggregation methods over the subdivisions were selected because they produced the highest predictive accuracy (R-square score) in our preliminary regression analysis. All our collected datasets are publicly available from [37].

### 2.2 Spatial analysis

All collected datasets were integrated into the census subdivision shapefile of Canada, downloaded from the Statistics Canada website [38]. Territorial subdivisions were excluded from the shapefile using the GeoPandas package of Python. Two spatial clustering methods, namely, local Gets Ord and Anselin local Moran's-I were used in ArcGIS Pro for cluster analysis.

To identify the most influential variables associated with the occurrence of HPAI, several regression approaches were evaluated, including XGBoost, Random Forest Regression (RFR), Geographically Weighted Regression (GWR), spatial regression, Ordinary Least Squares (OLS), and Generalized Additive Models (GAM). The number of HPAI cases in wild birds is treated as the dependent and the rest of the determinants as the independent variables in the regression analysis. The independent variables exhibited low to moderate correlations, with the highest correlation observed at 0.51 between mean minimum daily temperature and maximum CO concentration. Datasets such as human population, cloud fraction, Ozon (O3) concentration, sulfur dioxide (SO2) concentration, Ultra Violet (UV) index, and Net Primary Productivity (NPP) were removed from our dataset due to high correlation with other factors.

For non-tree based models, i.e. GWR, OLS, GAM, and spatial regression, multicollinearity was removed using a backward stepwise regression procedure [39]. However, an advantage of tree-based ensemble methods such as XGBoost and RFR is their robustness against multicollinearity among predictor variables, as their split-based structure can handle redundancy without affecting predictive performance. Five-fold cross-validation was used to assess model generalizability and to ensure that the selected models were not overfitted.

Moreover, spatial models, including GWR and spatial regression, internally compute spatial lags using the spatial weight matrix. In contrast, for the non-spatial regression models, such as XGBoost, RFR, OLS, and GAM the lagged independent variables were computed and inserted into the regression model as inputs. The spatial lag of each independent variable was calculated using the lag_spatial function of the Libpysal Python package. This function uses the spatial weight matrix of our shapefile to average each spatial value over its closest neighbors, according to eq. 1. Applying this method provides a locally-relevant value for each polygon (subdivision) by incorporating information from its neighbors.

(1) $$x_{\mathrm{lag},i}=\sum_{j=1}^{n}w_{\mathrm{ij}}x_{j}$$

Where, n is the total number of subdivisions, $x_{i}$ is the value of the specific independent variable for the i-th subdivision, and $w_{\mathrm{ij}}$ represents the spatial weight between subdivisions i and j. The lagged predictors exhibited slightly higher correlations, with the highest correlation being 0.69 between lagged mean minimum daily temperature and lagged maximum CO concentration. Correlation matrices of the independent variables before and after applying the spatial lag are available in Supplementary File 1.

The spatial weight matrix was constructed using several methods, including Queen, Rook, K-Nearest Neighbors (KNN), and distance band. For the KNN method, the optimal number of neighbors (k) that maximized the R^2^ score for our dataset was found to be k = 4. Among these, the distance band approach yielded the highest R^2^ score. This result is biologically reasonable, as the primary mechanism of H5N1 transmission in Canada is through migratory birds, which can traverse multiple subdivisions without being constrained by administrative boundaries. Unlike contiguity-based methods such as Queen or Rook, which only consider polygons sharing edges or corners, the distance band method accounts for spatial proximity based on geographic distance, better reflecting the ecological reality of bird-mediated disease spread. By applying a biologically relevant distance threshold, each subdivision incorporates contributions from others within the dispersal range of migratory birds, ensuring that the spatial lag of environmental predictors captures potential cross-district transmission pathways. We selected a threshold of 77 km for the distance band weights, corresponding to the maximum typical daily movement of migratory waterfowl during the breeding season [40]. This threshold captures plausible H5N1 transmission routes while avoiding unrealistic long-distance connections.

The significance of the correlation between the number of HPAI cases and environmental predictors was assessed using the bivariate Moran's I method. To account for multiple comparisons when examining the associations between HPAI cases in wild birds and multiple environmental variables, we applied the False Discovery Rate (FDR) adjustment using the Benjamini–Hochberg procedure. FDR controls the expected proportion of false positives among statistically significant results, which is especially important when testing several hypotheses simultaneously. In our analysis, we first calculated raw *p*-values from Moran's I correlations for each environmental variable, and then adjusted these *p*-values using the FDR method to reduce the risk of spurious associations while preserving statistical power. Variables with FDR-adjusted *p*-values below 0.05 were considered statistically significant [41].

Spatial regression was evaluated using various weight matrices (i.e. Queen, Rook, KNN, and distance band); however, GWR constructs its own location-specific weight matrices for each observation. These weights are determined using a distance-based kernel function, such as Gaussian, bi-square, or exponential, where observations closer to the target location receive higher weights and those farther away receive lower weights or are excluded entirely. In our analysis, selecting the Gaussian kernel function for the adaptive bandwidth, provided the best accuracy.

For all regression models, prior to regression model fitting, variables were scaled using the Min-Max normalization method to ensure consistency in variable ranges.

The SHapley Additive exPlanations (SHAP) method was applied to the XGBoost and RFR models to find the most important variables [42], [43]. SHAP offers several advantages that make it particularly suitable for interpreting complex, non-linear models. First, it remains reliable even in the presence of multicollinearity among predictor variables. Second, SHAP effectively captures non-linear relationships and feature interactions, which are common in ensemble tree-based algorithms such as XGBoost and RFR. Third, SHAP provides both global and local explanations, ensuring that feature attributions are consistent and locally accurate. Fourth, SHAP is theoretically grounded in cooperative game theory: each feature's contribution to a prediction is quantified using Shapley values, which offer a mathematically fair and interpretable distribution of importance across features. Together, these properties make SHAP a robust and interpretable tool for assessing variable influence in complex spatial models [42], [43]. SHAP returned almost the same important variables when used with both XGBoost and RFR on global (country) and local (subdivision) level analysis.

## 3 Results

### 3.1 Hot and cold spot detection

The hot and cold spots of the HPAI cases in wild birds have been detected using the Getis Ord (Fig. 1(a)) and Anselin local Moran's-I clustering (Fig. 1(b)). These figures have been analyzed and created using ArcGIS Pro. Both methods identified almost the same hot- and cold-spots of HPAI across Canadian subdivisions.

**Fig. 1:**
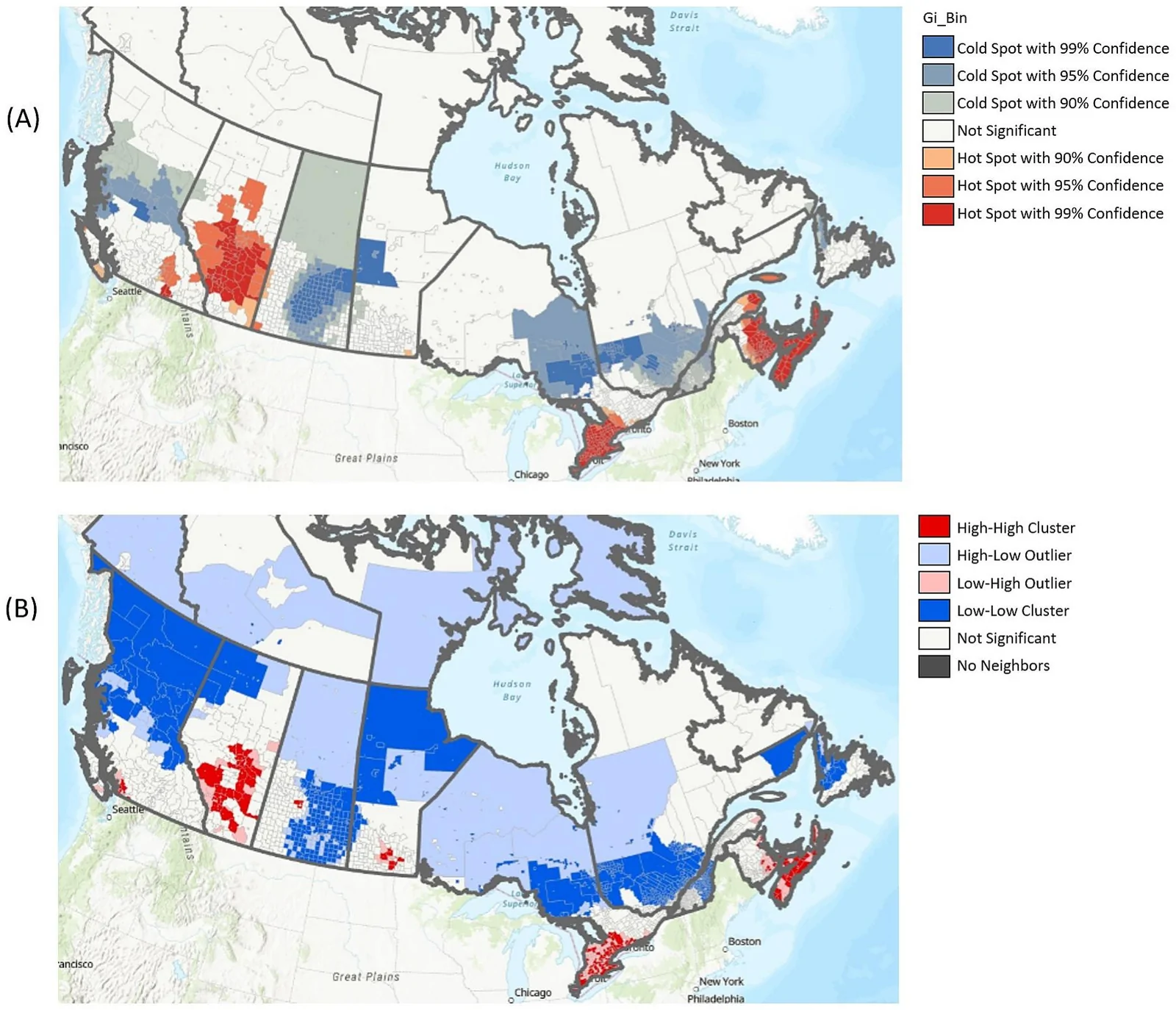
Hot and Cold spot detection of HPAI in wild birds in Canada on subdivision level using local (A) Getis Ord, and (B) Anselin Moran's-I. Colours indicate the cluster categories and statistical significance levels shown in each panel's legend. Hot spots represent spatial concentrations of relatively high HPAI case counts, whereas cold spots represent concentrations of relatively low case counts. Local Moran's I additionally distinguishes high–high and low–low clusters from spatial outliers (high–low and low–high). Only the provinces included in the study are shown.

As illustrated in Fig. 1, the methods identify southwest British Columbia, central and southern Alberta, southern Ontario, and the Atlantic provinces (i.e. New Brunswick, Nova Scotia, and Prince Edward Island) as the hot-spots, and central and northern British Columbia, southeastern Saskatchewan, central and northern Manitoba, central Ontario, and southern Quebec as the cold spots.

### 3.2 Model performance

Among the six regression methods evaluated, GAM, XGBoost, and RFR achieved acceptable R-square values, each explaining more than 40% of the variance (see Table A2 in Supplementary File 1). After addressing multicollinearity, nine predictors, namely, number of poultry farms, waterfowl population, mean minimum daily temperature, total rainfall, NO₂ concentration, GPP, water bodies, and wildfire, were retained, each with pairwise correlations below 0.5 (Supplementary File 1, Fig. A1) and a maximum Variance Inflation Factor (VIF) below 10 (Table 3) [44]. These predictors were used in the GAM model; however, multicollinearity was not removed for XGBoost and RFR because tree-based algorithms are intrinsically robust to correlated predictors. To ensure that the models were generalizable and not overfitted to the training data, five-fold cross-validation was applied. Table 1 compares the training accuracy and five-fold cross-validation accuracy across different spatial weight matrices.

**Table 1:** Comparing training accuracy and 5-fold cross validation accuracy of GAM, XGBoost, and RFR models with different weight matrices.

Model	Metric	Queen	Rook	KNN	Distance Band
Training accuracy					
**GAM**	**R-square**	0.177	0.172	0.165	**0.406**
	**RMSE**	0.04	0.041	0.042	**0.032**
	**MAE**	0.034	0.034	0.035	**0.032**
**RFR**	**R-square**	0.296	0.293	0.237	**0.545**
	**RMSE**	0.026	0.026	0.028	**0.025**
	**MAE**	0.022	0.022	0.027	**0.018**
**XGBoost**	**R-square**	0.481	0.477	0.440	**0.693**
	**RMSE**	0.024	0.024	0.025	**0.023**
	**MAE**	0.010	0.011	0.011	**0.008**
**5-fold cross validation accuracy**					
**GAM**	**R-square**	0.128	0.129	0.118	**0.319**
	**RMSE**	0.021	0.02	0.028	**0.001**
	**MAE**	0.014	0.014	0.022	**0.011**
**RFR**	**R-square**	0.156	0.149	0.129	**0.274**
	**RMSE**	0.04	0.041	0.042	**0.035**
	**MAE**	0.035	0.035	0.034	**0.024**
**XGBoost**	**R-square**	0.124	0.191	0.182	**0.312**
	**RMSE**	0.014	0.015	0.015	**0.034**
	**MAE**	0.013	0.012	0.012	**0.021**

Queen, Rook, KNN, and distance band refer to spatial weight matrices, used for finding lagged variables. Three metrics are used for evaluating the regression models, R^2^, RMSE, and MAE. These metrics are used to assess both the training and 5-fold cross validation accuracies. Higher R^2^ and lower RMSE/MAE generally indicate better predictive performance.

According to Table 1, among the different spatial weight matrices, the distance-band matrix provided the highest training and five-fold cross-validation accuracy. This is consistent with the migratory behavior of wild birds and the biological mechanisms underlying HPAI transmission. All models achieved moderate R-square values in five-fold cross-validation. The GAM model produced the highest cross-validation accuracy, and its training accuracy was the closest to its cross-validation accuracy, indicating that it was the most generalizable model. XGBoost showed slightly lower cross-validation accuracy and higher training accuracy, while the RFR model demonstrated acceptable training and cross-validation performance. Because all three models produced nearly identical rankings of feature importance, we retained them for subsequent analyses, which supports the robustness of our findings.

### 3.3 Spatial correlation analysis

Table 2 shows the Moran's I correlation between the predictors and the HPAI occurrences over subdivisions. As illustrated in Table 2, all indicators have a significant correlation, except for the number of poultry premises.

**Table 2:** The Moran's I correlation between the predictors and HPAI occurrences.

Variable	Moran's I correlation with HPAI cases	Raw *p*-value	FDR-adjusted p-value
NO2	- 0.109	0.001	0.001
Min daily temperature	0.101	0.001	0.001
CO	- 0.095	0.001	0.001
Elevation	- 0.087	0.001	0.001
Waterfowl population	0.041	0.005	0.0015
Total rain	0.038	0.001	0.001
GPP	0.037	0.005	0.0015
Wildfire	- 0.035	0.001	0.001
Tree coverage %	0.029	0.002	0.0016
Non-tree vegetation %	0.028	0.002	0.002
Water body	0.024	0.002	0.002
Non-vegetation %	- 0.021	0.008	0.0023
CH4	- 0.017	0.013	0.018
Number of poultry premises	0.007	Not Significant	Not Significant

This table presents the bivariate Moran's I statistics assessing spatial associations between each predictor and HPAI cases. Although the observed spatial associations were generally weak, the raw *p*-values indicated statistically significant associations for most predictors. To account for multiple hypothesis testing, FDR-adjusted *p*-values were also calculated. Following FDR correction, all predictors except the number of poultry premises remained statistically significant at the 0.05 level (adjusted *p* < 0.05). These findings suggest that all predictors except number of poultry premises exhibit statistically significant spatial associations with HPAI cases.

Although the number of poultry premises did not exhibit a statistically significant spatial correlation with H5N1 occurrences (Moran's I *p* = 0.099), it was identified by SHAP as one of the most important variables in XGBoost and RFR models.

### 3.4 Feature importance

The VIF of predictors after removing multicollinearity is displayed in Table 3. Moreover, Table 3 shows the Effective Degrees of Freedom (EDoF) and *p*-values of the predictors. Predictors with EDoF values near 1 are interpreted as approximately linear, whereas values substantially greater than 1 indicated nonlinear effects [45]. Since all predictors have an EDoF higher than 1 (Table 3), they have a nonlinear relation with the number of HPAI cases. This is supported by GAM smooth functions (Fig. 2) and the improved fit of the nonlinear XGBoost and RFR models relative to simpler linear approaches.

**Table 3:** Feature description in GAM analysis.

Predictor	VIF	EDoF	*P*-values
Waterfowl population	1.066	7.9	1.11e-16
Number of poultry premises	1.004	4.5	1.14e-9
GPP	4.65	5.1	1.47e-9
NO2	8.024	14.7	8.43e-7
Wildfire	1.367	5.6	8.04e-5
Minimum daily temperature	2.318	13.7	4.03e-3
Total rain	1.278	9.9	1.12e-1
Elevation	2.205	12.4	1.25e-1
Water bodies	3.109	9.7	9.89e-1
Intercept	–	0.0	1.09e-12

VIF values for all predictors were below the commonly used threshold of 10, suggesting that severe multicollinearity was unlikely to be a major concern in the model. The estimated EDoF were used to assess the shape of the smooth terms, with values substantially greater than 1 indicating nonlinear relationships and values close to 1 suggesting approximately linear relationships. Six predictors—waterfowl population, number of poultry premises, GPP, NO2, wildfire, and minimum daily temperature—had p-values below the 0.05 significance threshold, indicating statistically significant associations with HPAI cases within the fitted GAM. These results suggest that these predictors contribute significantly to explaining variation in HPAI cases.

**Fig. 2:**
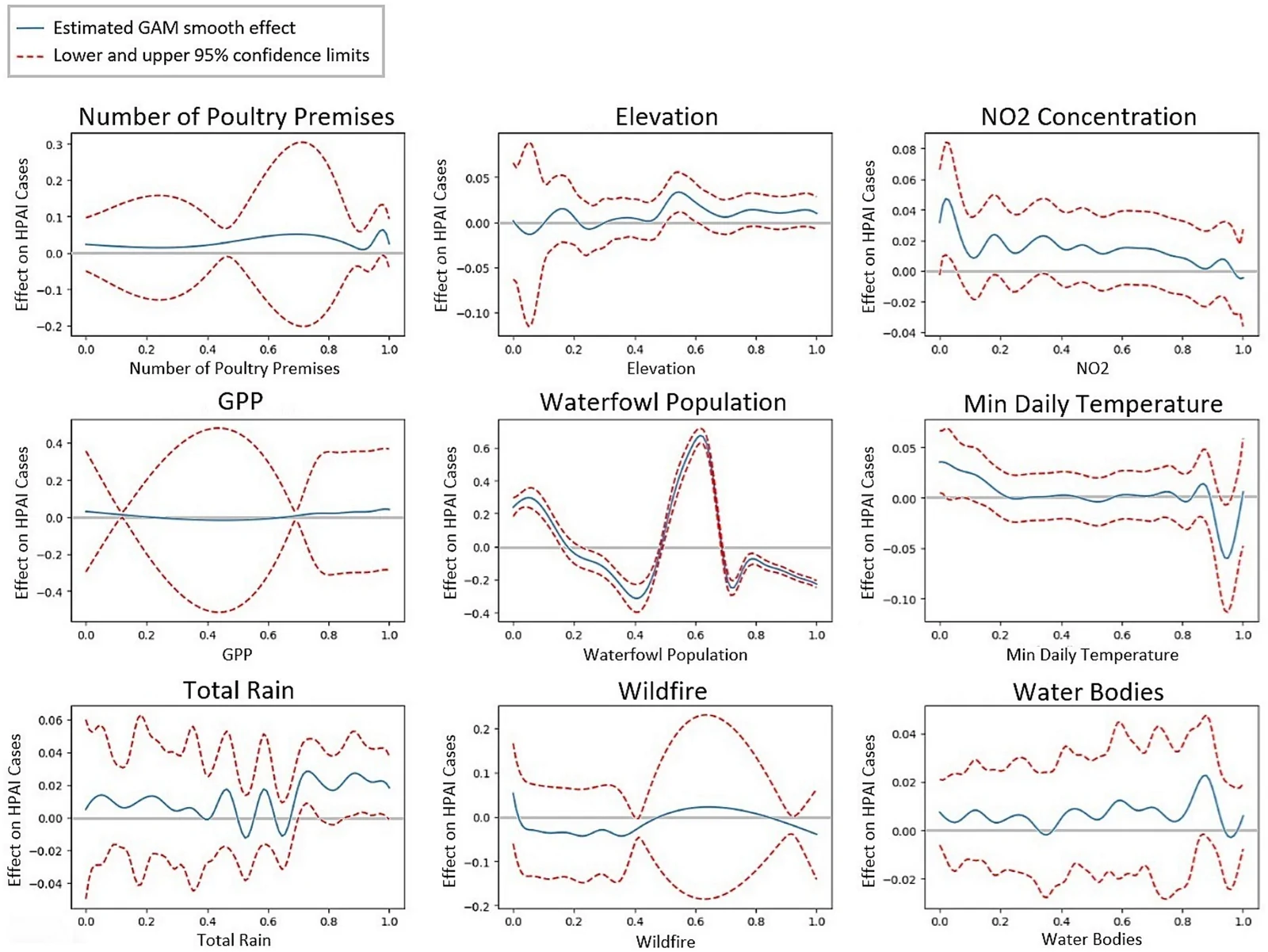
The non-linear relation between the number of HPAI cases and the predictors based on GAM smooth functions. Predictor values are Min–Max normalized, with 0 and 1 representing the minimum and maximum values, respectively, in the analyzed data. The solid blue line represents the estimated smooth effect, and the dashed red lines indicate the 95% confidence limits. The curves illustrate differences in the shape and direction of the estimated associations across predictors. These estimates represent model-based associations rather than causal effects; patterns near the extremes of the predictor ranges should be interpreted cautiously. (For interpretation of the references to colour in this figure legend, the reader is referred to the web version of this article.)

Table 3 shows that waterfowl population, the number of poultry premises, GPP, and air quality are the most influential predictors of HPAI case counts, whereas water bodies exhibit the weakest contribution. The EDoF further indicates that NO₂ concentration, mean minimum daily temperature, and elevation have the strongest nonlinear relationships with HPAI occurrence. Fig. 2 shows the GAM smooth functions for the number of HPAI cases and each predictor, which were visualized with 95% confidence intervals. The solid blue line represents the estimated nonlinear effect of the predictor on HPAI cases, while the dashed lines denote the associated 95% confidence bounds. This finding is consistent with previous studies that have reported non-linear patterns of avian influenza occurrence in Canada, particularly between the number of HPAI cases and mean minimum daily temperature [21].

On the national-level, Table 4 shows the mean SHAP values for different indicators over all subdivisions for XGBoost and RFR. In Table 4, the predictors have been ranked based on their importance.

**Table 4:** SHAP values for different indicators over all subdivisions for XGBoost and RFR.

XGBoost			RFR		
Rank	Variable	SHAP value	Rank	Variable	SHAP Value
1	Waterfowl population	0.005935	1	Waterfowl population	0.00631
2	Non-tree %	0.002500	2	Number of poultry premises	0.002454
3	Number of poultry premises	0.002495	3	Non-vegetation %	0.002204
4	GPP	0.001559	4	CH4	0.0019
5	CH4	0.00143	5	GPP	0.001844
6	NO2	0.00132	6	Non-tree %	0.001806
7	Non-vegetation %	0.001201	7	Tree coverage %	0.001589
8	Total rain	0.001175	8	NO2	0.001502
9	Wildfire	0.001148	9	CO	0.001486
10	Tree coverage %	0.001071	10	Wildfire	0.001484
11	Elevation	0.000874	11	Elevation	0.00127
12	CO	0.000855	12	Min daily temperature	0.001008
13	Min daily temperature	0.000338	13	Total rain	0.000407
14	Water body	0.000046	14	Water body	0.00031

The mean absolute SHAP values indicate that the XGBoost and RFR models identify largely similar predictors as the most important features, suggesting consistency in their assessment of feature importance.

Table 3 shows that both models produced a broadly consistent ranking of variable importance, with waterfowl population, vegetation parameters (i.e., GPP, non-tree, non-vegetation, and tree vegetation), number of poultry premises, elevation, and air quality (e.g., CH4 and NO2) emerging as the top predictors in both cases. Minor differences were observed in the relative ranking of some variables, e.g., NO2, CO, total rain, and minimum daily temperature, likely reflecting differences in how each model handles feature interactions. Overall, the similarity in ranking across two distinct modeling frameworks supports the robustness of the identified key predictors. Fig. 3 illustrates the most influential variables associated with HPAI occurrence at the local (subdivision) level. The results are based on the XGBoost model, which achieved the highest predictive accuracy. A corresponding visualization generated using the Random Forest Regression (RFR) model is provided in Supplementary File 1, Fig. A2(b), showing very similar spatial patterns.

**Fig. 3:**
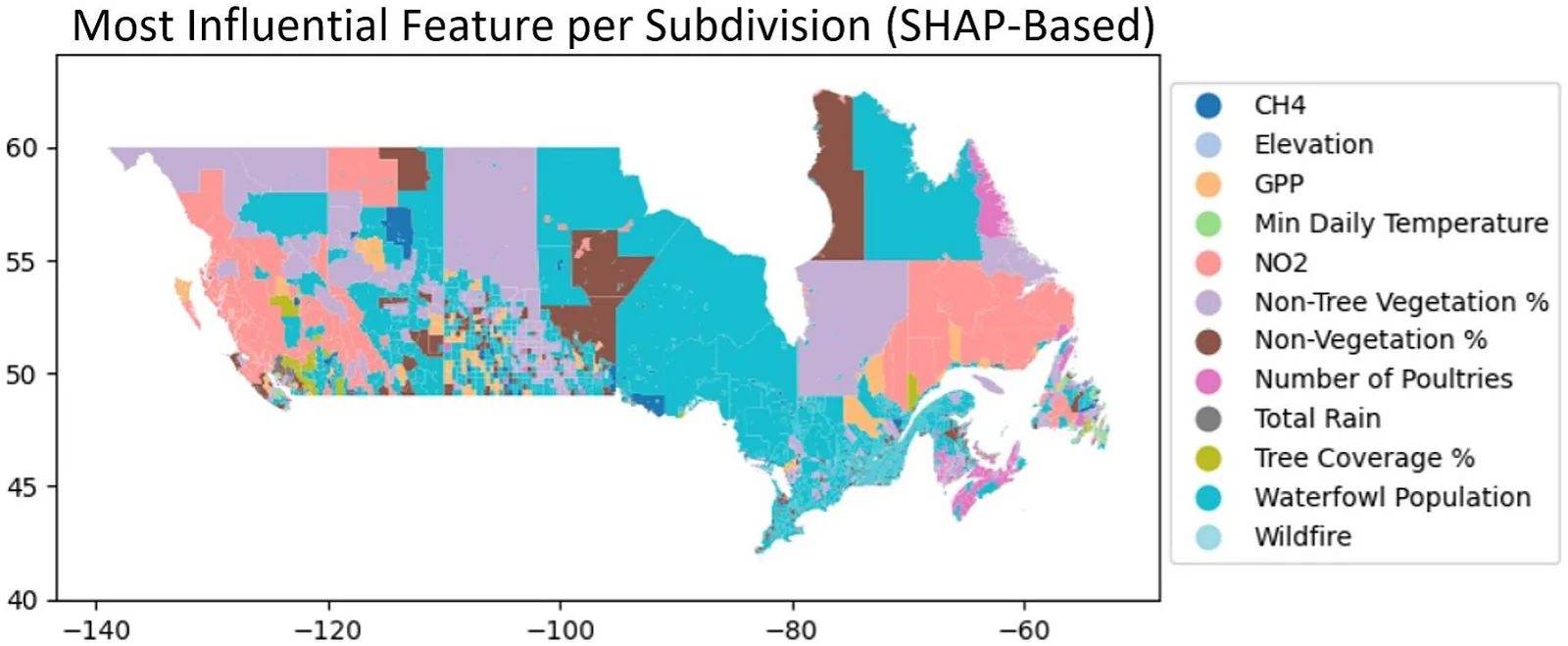
Most influential variables in each subdivision based on XGBoost. Each subdivision is colored according to the predictor identified as having the greatest SHAP feature importance for that subdivision. Colours correspond to the predictors listed in the legend. Waterfowl population is the predominant influential predictor across much of the study area, while other predictors are identified as most influential in geographically varying subsets of subdivisions. The map illustrates spatial variation in model feature importance and does not directly represent predicted HPAI case counts or causal effects.

A Chi-square goodness-of-fit test (*p* < 0.001) for both XGBoost and RFR revealed that certain variables were significantly more frequent as the most influential predictors of HPAI occurrence across subdivisions, indicating a non-uniform distribution of feature importance. Furthermore, a Moran's I value of 0.1059 (*p* = 0.001) for XGBoost, and 0.1761 (*p* = 0.001) for RFR confirmed the presence of significant spatial autocorrelation, suggesting that subdivisions dominated by similar top features tend to cluster geographically rather than being randomly distributed. Table 5 demonstrates the frequency of each variable across subdivisions.

**Table 5:** Frequency of variables contributing the most to HPAI occurrences across subdivisions based on XGBoost and RFR.

XGBoost		RFR	
Variable	Number of subdivisions	Variable	Number of subdivisions
Waterfowl population	3407	Waterfowl population	4214
Non-tree vegetation %	787	CH4	235
Non-vegetation %	315	Non-vegetation %	197
Number of poultry premises	177	Number of poultry premises	173
NO2	108	Non-tree vegetation %	105
GPP	103	GPP	72
CH4	65	Tree coverage %	21
Min Daily Temperature	51	NO2	19
Total rain	23	CO	11
Tree Coverage %	18	Total Rain	6
Elevation	2	Min daily temperature	2
Wildfire	1	Elevation	1
CO	0	Wildfire	1
Water bodies	0	Water bodies	0

For each XGBoost and Random Forest Regression (RFR) model, the counts indicate the number of subdivisions in which each variable was identified as the most influential predictor. Both models identified largely similar variables as the most frequently dominant predictors, indicating consistency in their identification of the leading predictors across subdivisions.

These results demonstrate that some environmental factors, such as waterfowl population density, GPP, vegetation coverage, air quality, and number of poultry premises are not only more influential overall, but also exhibit spatial patterns consistent with ecological processes underlying HPAI spread. These findings are also confirmed by RFR, as illustrated in Supplementary File 1.

At the local level, the subdivisions most affected by each predictor were identified and visualized. Fig. 4 illustrates the regions most strongly influenced by the key predictors based on the XGBoost model. Notably, the RFR model produced highly consistent spatial patterns (see Supplementary File 1). Detailed results for all predictors and both models (i.e. XGBoost and RFR) are provided in Supplementary File 1.

**Fig. 4:**
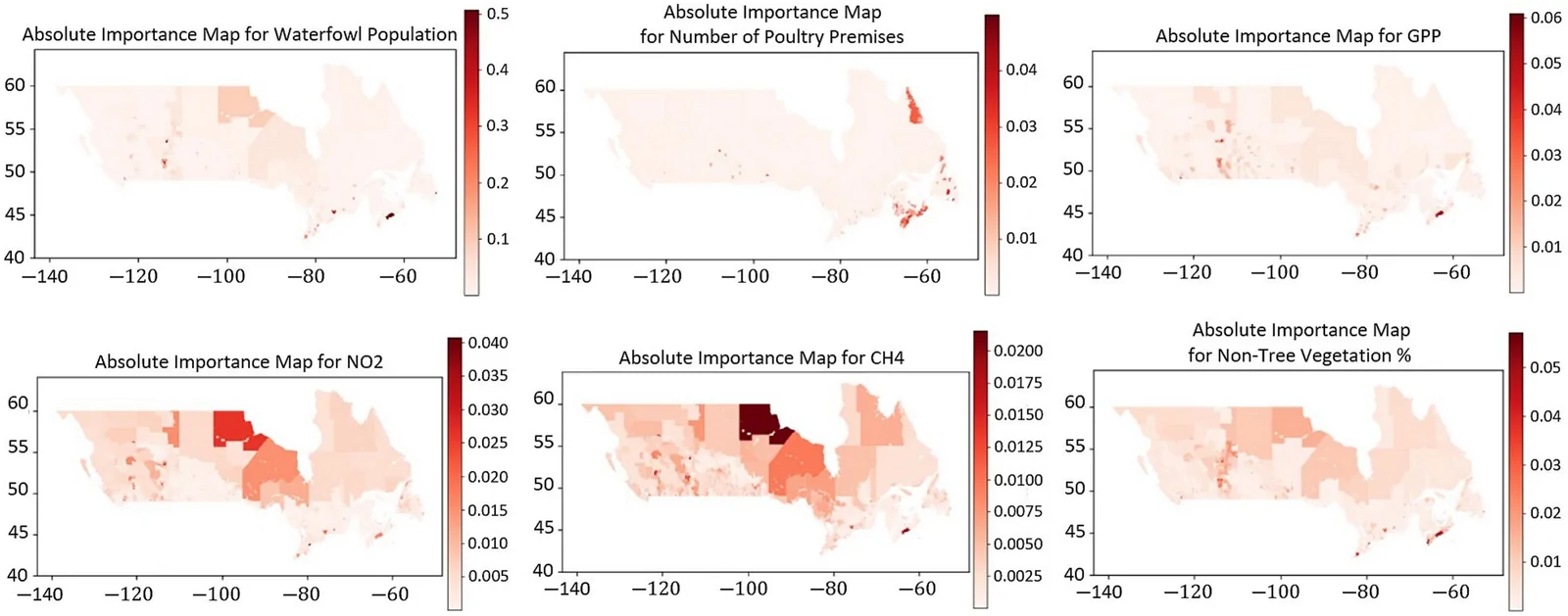
Subdivisions most strongly influenced by the key predictors based on XGBoost. The panels display the spatial distribution of importance values for key predictors identified by SHAP feature importance. The colour scale in each panel represents the corresponding importance measure, with larger values indicating greater model-attributed importance. These maps show where predictors contribute more strongly to model predictions.

## 4 Discussion

Identifying both hot and cold spots of HPAI occurrence is critical for effective national disease surveillance and management. Hot spots represent areas with significantly elevated disease incidence relative to their surroundings, indicating potential clusters of transmission or regions where biosecurity and control measures may require reinforcement. In contrast, cold spots reflect areas with consistently low or absent disease activity, which can highlight effective prevention practices, biosecurity protocols, or environmental conditions that limit viral spread. Recognizing both patterns allows for a more comprehensive understanding of spatial risk heterogeneity, guiding the officials and policy-makers in prioritizing surveillance resources, refining risk assessments, and supporting evidence-based policies for disease control and trade certification. Ultimately, simultaneous identification of high- and low-risk areas enhances the capacity to detect emerging threats while maintaining confidence in disease-free regions.

To address this need, the present study identified hot and cold spots of HPAI across Canada, and corresponding risk maps were generated to highlight areas of higher and lower disease risk.

All predictors had a significant Moran-I correlation with the number of HPAI cases, except for the number of poultry premises. However, the number of poultry premises was consistently identified by SHAP analysis as one of the most influential predictors in both XGBoost and RFR models. This discrepancy arises because correlation and spatial autocorrelation tests capture only linear or global relationships, whereas SHAP values reflect the variable's contribution within complex, nonlinear, and multivariate interactions. Therefore, poultry density likely influences HPAI risk in conjunction with other environmental and ecological factors, rather than through a simple direct association.

XGBoost and RFR were employed to capture the non-linear relationships between H5N1 occurrence and environmental predictors. Consistent with previous studies, our results indicate that waterfowl population and poultry density are among the most influential factors affecting HPAI occurrences [20].

However, our analysis expands upon existing research by incorporating a broader suite of environmental and ecological variables, specifically vegetation cover, air quality (particularly CH₄ concentrations), and elevation, which have not been previously examined in the Canadian context, were found as additional influential factors for HPAI occurrences.

Importantly, the observed association between poultry density and HPAI in wild birds does not necessarily imply that wild birds are attracted to poultry operations. Rather, it likely reflects the ecological and epidemiological connectivity between wild and domestic bird populations. Regions with high poultry density often overlap with habitats used by migratory waterfowl, facilitating potential cross-species transmission. Furthermore, these areas generally experience more intensive surveillance, which may contribute to higher detection rates in wild birds. Overall, the observed relationship likely represents a combination of habitat overlap, potential bidirectional transmission, and surveillance effects, rather than direct behavioral attraction of wild birds to poultry operations.

In addition to waterfowl population density and poultry density, we evaluated the influence of various climatic and environmental factors on HPAI occurrence, identifying GPP, vegetation, and air quality as important predictors. To our knowledge, such a comprehensive analysis of environmental determinants has not been previously conducted for Canada.

Although SHAP-based interpretation has some known limitations such as dependence on the trained model and potential sensitivity to correlated predictors, these issues are unlikely to substantially affect the present analysis. GAM smooth functions and EDoF show that there is a strong non-linear relation between the number of HPAI cases and the predictors. Moreover, the good performance of the XGBoost and RFR compared to other linear models support this nonlinear relationship. The consistency of GAM p-values and SHAP results across both tree-based models indicate that feature importance patterns are stable and robust. Multicollinearity was removed for GAM, in addition, for XGBoost and RFR, pairwise correlations among predictors were moderate, with maximum values of 0.51 for the original variables and 0.69 for their lagged counterparts, suggesting limited multicollinearity and reducing the risk of biased attributions. These conditions support the suitability of SHAP for interpreting non-linear relationships between predictors and HPAI cases in this study.

It is important to note that our findings are based on Canadian subdivisions and may not be directly generalizable to other regions with different ecological conditions or poultry production patterns. For instance, previous studies have highlighted region-specific drivers of HPAI: proximity to wetlands and bird orders in Denmark [13], and temperature in China [17]. These examples underscore that the relative importance of environmental and climatic factors can vary across geographic and ecological contexts.

In our analysis for Canada, water bodies were identified as the least important predictor of HPAI occurrence compared to other environmental variables both on national (Table 3) and local (Table 4) levels. One key reason is that water bodies are ubiquitous across the country, particularly in regions frequented by migratory waterfowl. Because this variable exhibits low spatial variability, the models have limited ability to distinguish high- versus low-risk areas based on water coverage alone. In other words, when nearly every subdivision contains a similar proportion of water bodies, machine learning models such as GAM, XGBoost, and RFR assign low importance to this predictor, as features with low variance generally contribute little to predictive differentiation. Furthermore, the behavior of wild birds in Canada differs from that in smaller or drier countries; migratory waterfowl often utilize a broad range of habitats, including large wetlands and agricultural mosaics, rather than being restricted to discrete water bodies [46]. By contrast, in countries with fewer or more spatially restricted water bodies, this feature serves as a stronger limiting factor and thus appears more influential in other studies.

To further examine the influence of HPAI infections in wild birds on domestic poultry and commercial bird farms, we conducted a complementary spatial analysis of HPAI cases in domestic poultry. We applied the same spatial clustering methods, Getis–Ord Gi* and Anselin Moran's I significance maps, to identify patterns of spatial aggregation. Fig. 5(A) presents the point-level case data extracted from the WAHIS dashboard [24], while Figs. 5(B) and 5(C) display the resulting hot and cold spot patterns generated using the Getis–Ord Gi* and Anselin Moran's I methods, respectively. As shown in Figs. 5(A) and 5(B), the spatial patterns for domestic poultry closely mirror those observed in wild birds: hotspots were concentrated in southern British Columbia, southern Alberta, and southern Ontario, whereas cold spots were observed in southeastern Saskatchewan, central British Columbia, central Manitoba, central Ontario, and portions of the Atlantic provinces. This substantial overlap in hotspot and cold-spot regions suggests a strong geographic correspondence in HPAI risk between wild and domestic bird populations.

**Fig. 5:**
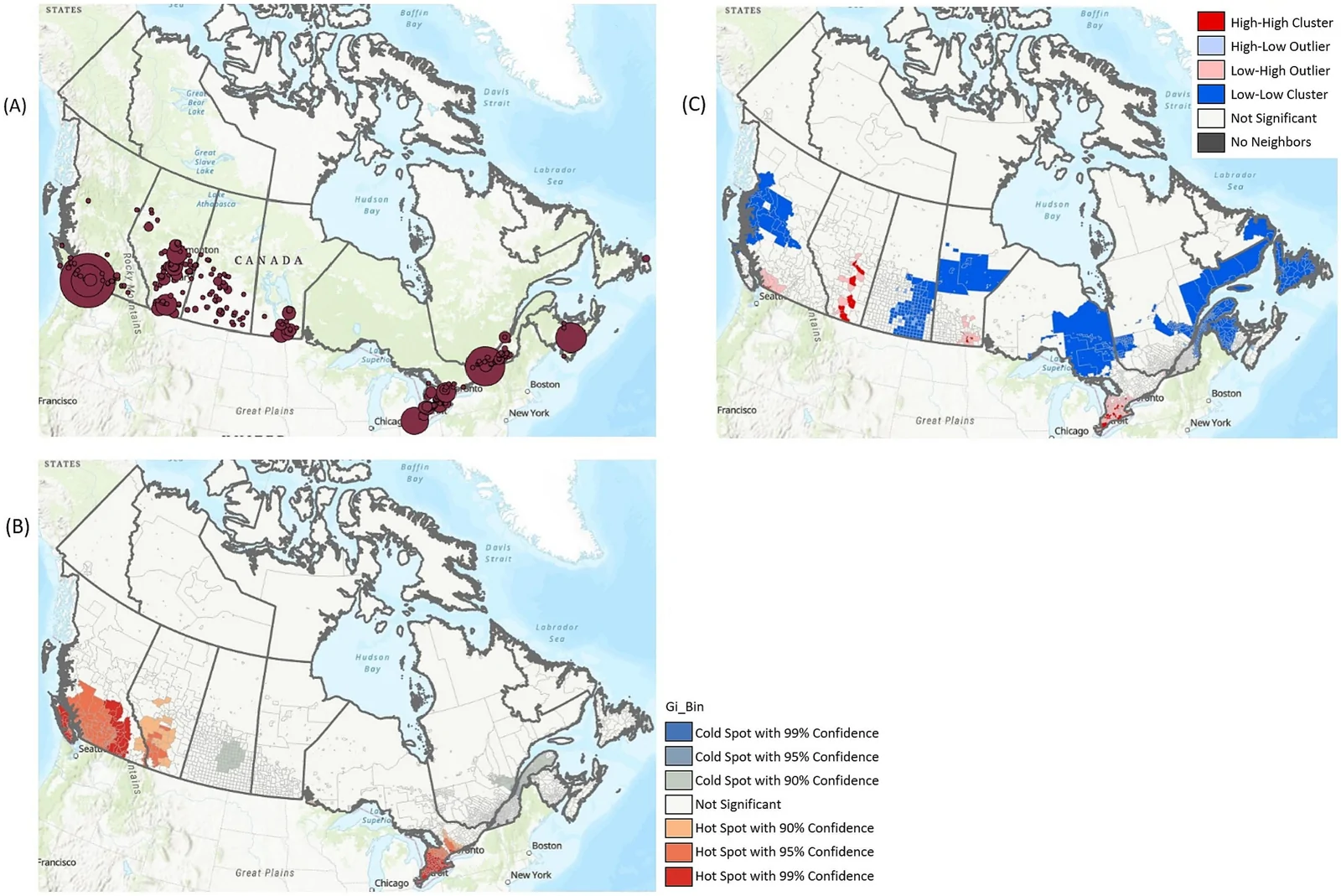
(A) point-level data of HPAI in domestic birds. The size of the points corresponds to the number of HPAI cases. (B) Hot and cold spots of HPAI in domestic birds using Getis-Ord Gi*. (C) Hot and cold spots of HPAI in domestic birds using Anselin Moran's I. Colours indicate the categories and significance levels shown in the respective map legends. The resulting patterns show geographic concentrations of domestic-bird HPAI cases, with several hotspots overlapping those identified in wild birds. This spatial correspondence suggests geographic overlap in HPAI occurrence.

The close alignment between hotspots in wild birds and domestic poultry suggests that environmental and ecological conditions driving HPAI circulation in wild bird populations may also elevate the risk of spillover into domestic flocks. From a policy and disease-management standpoint, this overlap provides important guidance for risk-based surveillance and intervention strategies. Poultry operations located within or adjacent to identified wild-bird hotspots may require heightened biosecurity measures, increased frequency of active surveillance, and prioritized resource allocation during outbreak periods. Conversely, cold spot regions may allow for a more proportionate, lower-intensity surveillance approach. Overall, the spatial concordance between wild and domestic cases underscores the interconnectedness of these populations and highlights the importance of integrated, landscape-based approaches to HPAI preparedness and response.

### 4.1 Limitations and future work

This study has several limitations that should be considered when interpreting the results. First, surveillance and reporting bias may influence the observed spatial patterns, as HPAI detections and waterfowl observations are more likely in areas with higher human activity and more intensive monitoring, potentially underrepresenting regions with low observer presence. Second, many environmental predictors used in the analysis are static or averaged over time, which may not fully capture seasonal or inter-annual variability in HPAI dynamics. Third, the spatial resolution of some datasets (e.g., climate or land cover) may mask fine-scale heterogeneity in habitats or outbreak locations, and resampling or interpolation could introduce additional uncertainty. Fourth, certain ecological and anthropogenic factors, such as small-scale water bodies, local biosecurity measures, or predator presence, were not included, which could influence disease dynamics. Finally, as climate change and land-use alterations continue to shift waterfowl habitats and migration patterns, the identified hot and cold spots may change over time, emphasizing the need for future research incorporating dynamic environmental and climate projections.

Future research could build upon this study by incorporating dynamic environmental and climate data to assess how seasonal variation, long-term climate change, and habitat shifts influence HPAI risk patterns. Integrating standardized or effort-corrected wildlife surveillance data could reduce reporting bias and improve the accuracy of spatial risk predictions. Additionally, combining fine-scale ecological, anthropogenic, and farm-level variables, such as local biosecurity measures, waterbody characteristics, and predator presence, may provide a more complete understanding of the drivers of disease spread. Finally, applying spatiotemporal models or predictive simulations could help forecast the evolution of hot and cold spots over time, supporting proactive disease management and targeted surveillance in both wild and domestic bird populations.

## 5 Conclusion

Two spatial cluster detection methods, namely, Getis-Ord and Anselin Moran's I, were applied to identify the hot and cold spots of HPAI across Canada. Both methods produced consistent results, revealing significant hotspots in the southwest of British Columbia, central and southern Alberta, southern Ontario, and the Atlantic provinces (New Brunswick, Nova Scotia, and Prince Edward Island). In contrast, cold spots were located in central and northern British Columbia, southeastern Saskatchewan, central and northern Manitoba, central Ontario, and southern Quebec. These results could inform risk-based active surveillance by prioritizing monitoring efforts in the identified hotspots. Focusing surveillance in southwest British Columbia, central and southern Alberta, southern Ontario, and the Atlantic provinces would allow earlier detection of HPAI outbreaks and more efficient allocation of resources. Conversely, regions identified as cold spots could be monitored less intensively, optimizing overall surveillance strategy.

Among the six regression models tested to identify key predictors of HPAI occurrence, only the GAM, XGBoost and RFR models achieved satisfactory performance, confirming the presence of strong non-linear relationships between HPAI case counts and environmental, agricultural, and climatic variables. SHAP analysis of both tree-based models identified the number of waterfowl, vegetation coverage, poultry density, GPP, and air quality (specifically methane concentration) as the most influential factors associated with HPAI occurrence at both national and subdivision scales.

These findings contribute valuable insights for improving avian influenza surveillance and prevention strategies in Canada. By pinpointing spatial hotspots and the most influential environmental and agricultural risk factors, this study provides evidence that can help public health authorities, animal health agencies, and policymakers target high-risk regions more effectively. The results also offer practical value for the poultry industry by informing risk-based biosecurity measures and guiding resource allocation for outbreak prevention and rapid response. More broadly, the integrated use of advanced spatial modeling and interpretable machine learning demonstrates a scalable framework that can support early warning systems, enhance One Health coordination, and strengthen preparedness for emerging zoonotic diseases.

## CRediT authorship contribution statement

**Zahra Movahedi Nia:** Writing – review & editing, Writing – original draft, Visualization, Validation, Software, Resources, Methodology, Investigation, Formal analysis, Data curation, Conceptualization. **Nicola Luigi Bragazzi:** Writing – review & editing, Validation, Supervision, Resources, Formal analysis, Data curation, Conceptualization. **Murray Gillies:** Writing – review & editing, Validation, Supervision, Resources, Formal analysis, Data curation, Conceptualization. **Emma Gardner:** Writing – review & editing, Validation, Supervision, Resources, Formal analysis, Data curation, Conceptualization. **Jude Dzevela Kong:** Writing – review & editing, Writing – original draft, Validation, Supervision, Software, Resources, Project administration, Methodology, Funding acquisition, Formal analysis, Data curation, Conceptualization.

## Funding statement

This research is funded by the NSERC Discovery Grant (Grant No. RGPIN-2022-04559). JDK acknowledges support from the NSERC Discovery Launch Supplement (Grant No. DGECR-2022-00454), the SSHRC New Frontiers in Research Fund – Exploratory (Grant No. NFRFE-2021-00879), Canada's International Development Research Centre (IDRC) (Grant No. 109981), as well as IDRC and the Foreign, Commonwealth and Development Office (FCDO) (Grant No. 110554–001) and Canada Research Chair in Community-Oriented Artificial Intelligence and Mathematical Modeling of Infectious Diseases (Award ID: CRC-2023-00234).

## Declaration of competing interest

The authors declare that they have no known competing financial interests or personal relationships that could have appeared to influence the work reported in this paper.

## Data Availability

The dataset of this research is available from [37].
