# Supplementary material for: Highly pathogenic avian influenza in Canada: Spatial analysis of ecological hot and cold spots and environmental drivers
Source: One Health. 2026 Oct 22;23:101630. doi: 10.1016/j.onehlt.2026.101630 (PMC13742085; doi:10.1016/j.onehlt.2026.101630)
Supplement: Supplementary file 1 [file mmc1.docx]

**Supplementary File 1:**

This document is a supplement to the following manuscript:

Highly Pathogenic Avian Influenza in Canada: Spatial Analysis of Hot and Cold Spots and Environmental Drivers

**Introduction**

Highly Pathogenic Avian Influenza (HPAI) outbreaks have risen globally in recent years, posing serious threats to public health, food security, and the poultry industry. Effective disease surveillance and prevention require understanding where outbreaks are most likely to occur and the environmental conditions that drive them. This study advances that goal by providing a comprehensive spatial analysis of HPAI in Canada, identifying high-risk regions and the key environmental and agricultural factors associated with outbreak patterns. The findings offer practical insights to support targeted surveillance, enhance biosecurity strategies, and guide evidence-based policy decisions within a One Health framework. In this document, additional information regarding the methodology and results is provided for transparency and reproducibility purposes.

1. **Waterfowl Population**

Table A1 summarizes the projects of FeederWatch^[1]^ that the bird population dataset was extracted from.

Table A1. The projects of FeederWatch that bird populations in Canada were collected from

| **Project Name** | **Number of Records** |
| --- | --- |
| Ebird Canada (Atlantic) [eBird Canada] | 41023 |
| Ebird Canada (BC) [eBird Canada] | 74700 |
| Ebird Canada (North) [eBird Canada] | 8575 |
| Ebird Canada (Ontario) [eBird Canada] | 254222 |
| Ebird Canada (Prairies) [eBird Canada] | 64962 |
| Ebird Canada (Quebec) [eBird Canada] | 104478 |
| Ebird Canada (sensitive records) [eBird Canada] | 201 |
| Newfoundland Breeding Bird Atlas (2020-2024) | 9863 |
| Newfoundland Breeding Bird species at risk (2020-2024) | 48 |
| Ontario Breeding Bird Atlas (2021-2025) | 173607 |
| Ontario Breeding Bird Atlas, species at risk (2021-2025) | 4912 |
| Quebec Breeding Bird Atlas (northern project) | 600 |
| Saskatchewan Breeding Bird Atlas (2017-2021) | 5467 |
| Saskatchewan Breeding Bird Atlas, species at risk (2017-2021) | 107 |
| Alberta Bird Records [NatureCounts] | 3512 |
| Alberta Breeding Bird Atlas (2000-2005) | 15834 |
| Manitoba Breeding Bird Atlas (2010-2014) | 9303 |
| **Total** | **771414** |

The dataset included various bird species. Large Language Models (LLM), particularly ChatGPT were used to find a list of waterfowl species of the dataset. Next the dataset was filtered for the waterfowl species, which are listed below.

Mallard, northern shoveler, ruddy duck, canvasback, ring-necked duck, lesser scaup, greater scaup, bufflehead, wood duck, common eider, king eider, long-tailed duck, harlequin duck, american black duck, cinnamon teal, blue-winged teal, green-winged teal, surf scoter, black scoter, american wigeon, northern pintail, muscovy duck, Aythya, diving duck, scaup, ring-necked, redhead, canvasback, duck sp., duck, dabbling duck sp., dabbling duck, blue-winged/cinnamon teal (hybrid/uncertain sp.), blue-winged, cinnamon teal, american wigeon x mallard (hybrid), wigeon

2. **Correlation Matrix of the Predictors**

Figure A1(a) shows the correlation matrix of original predictors and Figure A1(b) shows the correlation matrix of lagged predictors. The methodology for finding the lagged variables is already explained in the manuscript.

(a) (b)

Figure A1. Correlation matrix of the (a) original predictors and (b) lagged predictors

3. **Model Selection**

Six models, namely, Ordinary Least Square (OLS), Generalized Additive Model (GAM), spatial regression, Random Forest Regression (RFR), XGBoost, and Geographically Weighted Regression (GWR) were used for regression analysis. Table A2 compares different regression models with different spatial weight matrices using R-square score, Root Mean Square Error (RMSE), and Mean Absolute Error (MAE).

Table A2. Comparing various regression models with different weight matrices

| **Model** | **Metric** | **Queen** | **Rook** | **KNN** | **Distance Band** |
| --- | --- | --- | --- | --- | --- |
| **OLS** | **R-square** | 0.19 | 0.19 | 0.26 | 0.268 |
|  | **RMSE** | 0.041 | 0.041 | 0.041 | 0.036 |
|  | **MAE** | 0.014 | 0.014 | 0.013 | 0.013 |
| **GAM** | **R-square** | 0.177 | 0.172 | 0.165 | **0.406** |
|  | **RMSE** | 0.04 | 0.041 | 0.042 | **0.032** |
|  | **MAE** | 0.034 | 0.034 | 0.035 | **0.032** |
| **Spatial regression** | **R-square** | 0.2859 | 0.2859 | 0.253 | 0.296 |
|  | **RMSE** | 0.036 | 0.036 | 0.037 | 0.037 |
|  | **MAE** | 0.011 | 0.011 | 0.014 | 0.012 |
| **RFR** | **R-square** | 0.296 | 0.293 | 0.237 | **0.545** |
|  | **RMSE** | 0.026 | 0.026 | 0.028 | **0.025** |
|  | **MAE** | 0.022 | 0.022 | 0.027 | **0.018** |
| **XGBoost** | **R-square** | 0.938 | 0.938 | 0.973 | **0.693** |
|  | **RMSE** | 0.024 | 0.024 | 0.025 | **0.023** |
|  | **MAE** | 0010 | 0.011 | 0.011 | **0.008** |
| **Method** | **Metric** | **Adaptive Bandwidth Weights with Gaussian Kernel function** | | | |
| **GWR** | **R-square** | 0.372 | | | |
|  | **RMSE** | 0.033 | | | |
|  | **MAE** | 0.012 | | | |

This result shows that the highest accuracy is obtained with GAM, XGBoost and RFR.

4. **Feature Importance on Local Level**

Figure A2 shows the most influential variables across subdivisions based of Random Forest Regression (RFR).

Figure A2. Most influential variables across subdivisions according to RFR

Moreover, Figure A3 (a) illustrates the subdivisions most influenced by each feature based on the XGBoost model, while Figure A4 (b) that for the Random Forest Regression model.

XGBoost: RFR:

RFR

XGBoost

RFR

XGBoost

RFR

XGBoost

RFR

XGBoost

RFR

XGBoost

RFR

XGBoost

RFR

XGBoost

RFR

XGBoost

RFR

XGBoost

RFR

XGBoost

RFR

XGBoost

RFR

XGBoost

RFR

XGBoost

RFR

XGBoost

**Conclusion**

This study enhances understanding of the spatial dynamics and environmental drivers of HPAI in Canada, offering a foundation for more targeted and efficient disease surveillance. The findings can help policymakers and the poultry industry strengthen prevention, biosecurity, and early warning efforts to reduce future outbreak risks.

1. <https://naturecounts.ca/nc/default/datasets.jsp?code=PFW>
